# Exploring links between oral health and infective endocarditis

**DOI:** 10.3389/froh.2024.1426903

**Published:** 2024-12-02

**Authors:** Joseph Luke Falconer, Ronak Rajani, Vitaliy Androshchuk, Amieth Yogarajah, Rebecca A. Greenbury, Ayden Ismail, Natasha Oh, Luigi Nibali, Eleanor M. D’Agostino, Vanessa Sousa

**Affiliations:** ^1^ Centre for Host-Microbiome Interactions, Faculty of Dentistry, Oral and Craniofacial Sciences, Kings College London, London, United Kingdom; ^2^ Department of Periodontology, Guy’s and St Thomas’ NHS Foundation Trust, London, United Kingdom; ^3^ Department of Cardiology, Guy’s and St Thomas’ NHS Foundation Trust, London, United Kingdom; ^4^ Department of Anaesthesia, King’s College Hospital NHS Foundation Trust, London, United Kingdom; ^5^ Ealing Hospital, London North West University Healthcare NHS Trust, London, United Kingdom; ^6^ Unilever Oral Care, Bebington, United Kingdom

**Keywords:** oral health, endocarditis, diagnostics, microbiome, periodontal

## Abstract

Infective endocarditis (IE) is a bacterial infection of the heart's inner lining. A low incidence rate combined with a high mortality rate mean that IE can be difficult to treat effectively. There is currently substantial evidence supporting a link between oral health and IE with the oral microbiome impacting various aspects of IE, including pathogenesis, diagnosis, treatment, and mortality rates. The oral microbiome is highly diverse and plays a crucial role in maintaining oral health by providing protective functions. However, when dysbiosis occurs, conditions such as periodontal or peri-implant disease can arise, offering a pathway for bacteraemia to develop. The role of the oral microbiome as a coloniser, facilitator and driver of IE remains to be uncovered by next-generation sequencing techniques. Understanding the dysbiosis and ecology of the oral microbiome of IE patients will allow improvements into the diagnosis, treatment, and prognosis of the disease. Furthermore, an increased awareness amongst those at high-risk of developing IE may encourage improved oral hygiene methods and lower incidence rates. This narrative review examines current findings on the relationship between oral health and IE. It draws from key studies on both topics, with manuscripts selected for their pertinence to the subject. It highlights the link between the oral microbiome and IE by exploring diagnostic techniques and treatments for IE caused by oral commensals.

## Overview of infective endocarditis and the oral microbiome

Infective endocarditis (IE) is a disease characterised by inflammation or infection of the endocardial surface of the heart. It can affect native and prosthetic heart valves as well as cardiac implantable electronic devices (CIEDs) such as pacemakers, defibrillators and cardiac resynchronisation therapy devices. Although relatively rare, with an annual age- and gender-standardised incidence of 3–10 cases per 100,000 inhabitants (1), it is recognised as a major worldwide health challenge with a mortality of up to 30% at 30 days. Patients with IE are predominantly male and generally older, with a peak incidence between the ages of 70 and 80 years (2). The bacterial port of entry is usually via the transcutaneous, oral or respiratory routes, and less commonly through the gastrointestinal or genitourinary tracts. *Staphylococcus aureus* is now the predominant causative microorganism of IE in the developed world, accounting for 30% of all cases, followed by *Streptococci* and *Enterococci* (3). In all cases of suspected IE, careful clinical and cardiac imaging evaluation is required to establish an early diagnosis and to enable prompt treatment with antimicrobial therapy and/or cardiac surgery before progressive cardiac involvement or systemic complications ensue.

For both native valve IE and prosthetic valve IE cases, bacteraemia is a prerequisite (4). Under healthy conditions, the valvular endothelium is resistant to bacterial colonisation, and thus a disruption of the valvular surface is necessary for bacterial attachment (5, 6). Once bacterial colonisation occurs, the bacteria form biofilms —complex communities embedded in a matrix of secreted macromolecules (7). Biofilm formation not only facilitates immune evasion but also contributes to suboptimal antibiotic therapy by creating a protective barrier that impedes antibiotic penetration. Pathogens from the oral microbiome can enter the bloodstream, attach to damaged cardiac tissue, and initiate an inflammatory response, ultimately leading to the characteristic lesions of IE (8).

Good oral health contributes to both quality of life and systemic health. The oral microbiome has co-evolved with humans, forming an ecosystem which is important for maintaining health when in homeostasis (9). Within the spectrum of IE, the oral microbiome has been heavily implicated in the causation of IE (10, 11). Recent studies indicate that common dental procedures (including non-surgical) may result in bacteraemia of typical oral commensals in 60%–80% of patients (12). Mechanical plaque control through toothbrushing is well established (13). However, given that the milder forms of periodontitis occur in up to 50% of the general population (14), even simple toothbrushing may increase the risk of transient bacteraemia (15, 16). The healthy myocardial endocardium is generally resistant to the bacteraemia arising from daily activities such as chewing and tooth brushing (17). However, endothelial injury arising from almost any type of congenital or degenerative structural heart disease can increase the risk of IE (8). Other factors that may be associated with endothelial bacterial adherence and IE include systemic inflammatory diseases, such as diabetes mellitus, human immunodeficiency virus (HIV), rheumatoid arthritis, systemic lupus erythematosus, intravenous drug use, previous IE and rheumatic heart disease (18). Widespread medical advances during the 20th and 21st centuries have played a substantial role in shaping the current patient demographics and microbiology of IE. For example, an increased prevalence of prosthetic valves, CIEDs, long-term intravenous lines and invasive procedures now form the principal risk factors. As a result, healthcare-related IE (hospital-acquired and outpatient-acquired) accounts for up to one-third of all cases of IE in contemporary cohorts (19).

Despite advances in diagnostic imaging, antibiotic therapies and surgical or percutaneous valvular treatment options, the prognosis for IE remains poor. Even with optimal therapy, IE is associated with high in-hospital mortality rate of up to 25% (20, 21). Complications of IE related to haemodynamic instability, embolisation, septic abscesses and neurological events are amongst the most devastating, and have a significant effect on increasing mortality (22, 23). Early surgery in high-risk patients with *Staphylococcus aureus* IE (24) can be further complicated with IE relapses and postoperative valvular dysfunction (25). As a result, even after the acute phase, the risk of mortality for IE remains high, with survivors affected by increased morbidity and reduced quality of life (26). Therefore, it is imperative that clinicians have a high index of suspicion for the diagnosis of IE. This is particularly pertinent in patients that present with relevant symptoms, risk factors and typical microorganisms. The European Society of Cardiology (ESC) guidelines provide an evidence-based framework for the best diagnostic and therapeutic approach to managing patients with IE (27).

## Current diagnostic methods for infective endocarditis

### The modified Duke criteria

Reaching an early and accurate diagnosis of IE requires a combination of clinical suspicion, microbiology analysis and cardiac imaging with evidence of IE-related damage. Evaluation of the patients with suspected IE relies on the modified Duke criteria (MDC) (28), which integrates these clinical domains and classifies them into minor and major criteria. A definite diagnosis of IE requires two major, one major and three minor, or five minor criteria to be met (29). It should be noted that the MDC has lower sensitivity for patients with CIED-related IE, right-sided IE, prosthetic valve endocarditis (PVE) and blood culture negative endocarditis (BCNE) (30). For this reason, the MDC should be used as a guide for assimilating the clinical and laboratory findings to confirm the diagnosis, rather than a replacement for clinical judgement (31).

### Microbiology

Blood cultures (BC) are the first and essential diagnostic tool for identifying continuous bacteraemia in IE (32, 33). Three sets of BCs should be drawn at 30-minute intervals from separate sites and using aseptic technique before patients are started on antibiotics (32). The standard practice of incubation for BC is 5–7 days at 35–37°C, which achieves bacteraemia detection rate of 96%–98% (32, 34). Around 10%–20% of patients with IE have negative BC results, creating diagnostic uncertainty (10). This can arise due to antibiotic treatment before BCs, infection with fastidious fungi or characteristically slow-growing bacteria (*Haemophilus* species; *Aggregatibacter* species; *Cardiobacterium hominis; Eiknella corrodens*; and *Kingella species—*HACEK organisms) (32) or an alternative diagnosis. Improved practices for BC sampling, modification of loading delay, BC preincubation and extended BC incubation can help to improve the diagnostic yield (32, 35, 36). Direct identification of specific organisms can be achieved using other techniques, including serological testing, broad-range polymerase chain reaction (PCR) and mass spectroscopy (37). Recently, metagenomic techniques have been considered to offer non-invasive diagnosis and biomarker discovery (38). Metagenomic diagnosis for IE could provide a faster alternative diagnostic tool, improving outcomes for patients in the future. A recent systematic review highlighted that current metagenomic methods have satisfactory diagnostic performance and yield an overall detection rate higher than some conventional methods (39).

### Cardiac imaging

The MDC defines three major imaging criteria for IE: abscess, vegetation and new dehiscence of prosthetic valve (28). Transthoracic echocardiography (TTE) remains the cornerstone of imaging for the initial assessment of suspected IE (27). Transoesophageal echocardiography (TEE) is recommended when TTE is equivocal or non-diagnostic, when complications are suspected, or when intracardiac device leads are present. Cardiac computed tomography (CT) is an important adjunctive imaging modality for use when the diagnostic performance of echocardiography is affected by artifacts or when TEE is contraindicated altogether (40). Cardiac CT has high spatial resolution, which can help to delineate paravalvular anatomy for complications (the extend of abscesses, fistulae or mycotic aneurysms) and plan surgical interventions. Compared with CT, TEE has improved temporal resolution and is therefore more useful for identifying small IE vegetations (<10 mm) and valvular incompetence (41). Additional diagnostic value can be provided by combining CT with metabolic imaging using 18F-fluorodeoxyglucose positron emission tomography (^18^FDG-PET) or leukocyte scintigraphy [radiolabelled leukocyte single-photon emission computed tomography (SPECT)], which can help to show areas of increased metabolic activity or inflammation in the regions of suspected IE (42). Awareness of the available imaging modalities and the relative strengths and weaknesses of each technique facilitates assessment of patients with suspected IE.

## Management of infective endocarditis

Effective management of IE requires a multidisciplinary input from infectious disease specialists, cardiologists and cardiothoracic surgeons. Empirical antimicrobial therapy can be started after the BCs are taken, with further targeted amendments based on BC results, resistance patterns, infection severity and the presence of prosthetic material. A combination of antibiotics is usually needed for up to 6 weeks to eradicate the culprit organism (43). Prolonged regimens are required to obtain a sustained bactericidal effect given that the infections of the valvular apparatus are usually difficult to reach by the host immune system and antibiotics. Stable patients who are responding well to treatment may be considered for an early shift from intravenous to oral antibiotics for the remainder of the course (44). Emerging evidence also suggests that effective courses of antibiotics with the duration as short as two weeks can be given to some IE patients with no complications and highly susceptible microbial strains (45). Prompt referral for surgery is indicated for significant valve dysfunction with heart failure, embolic complications and persistent infection (46–48). Surgery aims to restore normal valve function and to resect all infected tissue. Valvular repair or replacement using either bioprosthetic or mechanical valves may be considered (49).

## Discussion

### Oral microbiome, oral health and infective endocarditis

The oral microbiome is one of the most complex ecosystems within the human body and plays an important role in physiological, metabolic, immunological and systemic health (50). The oral cavity itself is comprised of a multitude of distinct habitats created by the periodontal pocket, gingivae, tongue, teeth, cheek, palate and saliva. These environments provide an ideal medium for more than 700 known bacterial species to reside, with an ever increasing number being continuously discovered using metagenomic profiling techniques (9, 51). As a consequence, the oral cavity remains an important portal of entry for microorganisms into the blood stream that are heavily implicated in the causation of infective endocarditis (50).

Recent studies indicate that common dental procedures such as flossing, toothbrushing, and chewing may result in transient bacteraemia involving common oral commensals (15, 52). More invasive procedures, such as endodontic treatments, tooth extractions, caries removal, and periodontal or apical surgery, can also induce gingival or mucosal trauma and subsequent bacteraemia (53). Several factors, including diet, smoking, alcohol consumption, stress, and antibiotic therapy, are associated with changes in the oral microbiota, potentially disrupting the balance between commensal and pathogenic organisms (54). This imbalance, known as dysbiosis, is a major driver of periodontal disease and tooth loss (55, 56). Chronic inflammation and pathophysiological destruction of the gingival tissue and bone allows oral pathogens to cause bacteraemia with less resistance, contributing to systemic inflammation (57).

The relationship between oral dysbiosis, periodontal disease, and IE remains incompletely understood. However, monitoring changes in the oral microbial composition of IE patients is essential for developing diagnostic tools aimed at preventing periodontal disease and mitigating the systemic sequelae associated with increased bacteraemia. Conventional periodontal diagnostic methods, such as clinical examinations and imaging, are effective in detecting periodontal disease, which, if left untreated, can increase the risk of cardiovascular disease (58). Although these methods do not directly diagnose IE, they play a crucial role in managing oral health and reducing the risk of bacteraemia. Advanced techniques, such as microbial testing, are increasingly valuable in assessing the risk of systemic infections caused by specific oral pathogens. Early management of periodontal and peri-implant diseases using these diagnostic tools can significantly reduce the risk of bacteraemia and systemic complications, including IE, particularly in high-risk patients. The treatment of periodontitis and management of peri-implant diseases (59, 60) including professional mechanical plaque removal, are essential for reducing microbial load and controlling inflammation. By maintaining the integrity of the oral epithelium, these interventions help to reduce gingival inflammation and bleeding, prevent periodontal disease progression, and lower the risk of systemic infections.

It is important to note that current guidelines, including those from the American Heart Association (AHA) and the European Society of Cardiology (ESC), recommend antibiotic prophylaxis (AP) only for high-risk patients undergoing invasive dental procedures (43). In contrast, the National Institute for Health and Care Excellence (NICE) in the United Kingdom has advised the discontinuation of routine AP for IE prevention in patients with heart conditions undergoing dental procedures [NICE guideline CG64, 2008; updated in 2015 (61)]. Despite these differing recommendations, uncertainty remains about the precise relationship between the oral microbiome and IE, highlighting the need for further research (11). This underscores the importance of good oral hygiene and regular periodontal care as primary preventive measures in managing patients at risk of IE.

### Common causative oral transient bacteria of IE

Saliva is abundant with bacteria, containing up to 10^8^ microbes per millilitre (62). Under poor oral health conditions, the concentration of certain periodontal pathogens can increase. Several of these microorganisms have been identified to be causative of IE (Table 1). Oral *Streptococci* are historically associated with the development of IE and are found in around 20% of all cases (21, 118). Viridian Group *Streptococci* (VGS) represent the most common species linked with IE. Patients affected by periodontitis have twice the mortality rate when IE is caused by VGS rather than other bacteria (119). Periodontitis has been shown to enhance bacteraemia caused by VGS (120). Although there is a clear link, taxonomical classification between VGS species has proven difficult due to their ability to take up free DNA from the external environment. VGS are categorised into 5 groups: the *S. salivarius* group, the *S. mitis* group, the *S. mutans* group, the *S. sanguinis* group and the *S. anginosus* group. The most common causative pathogen of the VGS species is *S. sanguinis*, making up over 30% of all VGS IE cases (121, 122). Under normal conditions it is possible for *S. sanguinis* to form a monospecies oral biofilm. However, in the bloodstream *S. sanguinis* can adhere to the circulating platelets and submucosal collagens at the sites of valvular damage, which can predispose to the formation of IE vegetations (123).

**Table 1:** IE-associated microorganisms and oral pathogens in human saliva and subgingival plaque samples (11, 63–72).

Microorganism (Genera)	Microorganism (Species)	Presence in saliva	Presence in plaque	Associated oral pathology	IE association	Reference
*Staphylococcus*	*Staphylococcus aureus*	Shared	Shared	Periodontitis, Peri-implantitis	Leading cause of IE	(73) (74) (75)
	*Staphylococcus epidermidis*	Shared	Shared	Caries, periodontitis, peri-implantitis	Common cause of IE	(76) (77) (78)
	*Staphylococcus warneri*	Shared	Shared	Periodontitis	Rare cause of IE	(79) (80)
	*Staphylococcus hominis*	Shared	Shared	No known	Rare cause of IE	(81)
	*Staphylococcus capitis*	Shared	Shared	Periodontitis	Rare cause of IE	(82) (83)
	*Staphylococcus lugdunensis*	Unique	Absent	Aids in acute oral infection	Rare cause of IE	(84) (85)
	*Staphylococcus haemolyticus*	Shared	Shared	Periodontitis	Rare cause of IE	(82) (86)
*Streptococcus*	*Streptococcus mitis*	Shared	Shared	Dental caries	Common cause of IE	(87) (88)
	*Streptococcus sanguinis*	Shared	Shared	Dental caries	Common cause of IE	(87) (89)
	*Streptococcus salivarus*	Shared	Shared	No known	Moderate cause of bacteraemia, rare cause of IE	(90)
	*Streptococcus anginosus*	Absent	Unique	Dental abscesses, periodontitis	Common cause of bacteraemia, rare cause of IE	(91) (90)
	*Streptococcus pneumoniae*	Shared	Shared	Periodontitis	Common cause of bacteraemia, rare cause of IE	(92) (90)
	*Streptococcus mutans*	Shared	Shared	Dental caries, periodontitis	Leading cause of IE	(87) (93) (90)
	*Streptococcus gordonii*	Shared	Shared	Periodontitis	Common cause of IE	(94) (90)
	*Streptococcus cristatus*	Absent	Unique	No known	Infrequent cause of bacteraemia	(95)
	*Streptococcus oralis*	Shared	Shared	Periodontitis	Common cause of IE	(96) (90)
	*Streptococcus parasanguinis*	Shared	Shared	Periodontitis	Common cause of IE	(94) (90)
*Enterococcus*	*Enterococcus faecalis*	Shared	Shared	Periodontitis, peri-implantitis, caries	Leading cause of IE	(97) (98)
*Other*	*Aggregatibacter aphrophilus*	Shared	Shared	No known	Rare cause of IE	(99)
	*Neisseria elongata*	Shared	Shared	No known	Rare cause of IE	(100)
	*Abiotrophia defective*	Shared	Shared	No known	Rare cause of IE	(101)
	*Granulicatella adiacens*	Shared	Shared	Periodontitis	Rare cause of IE	(94) (102)
	*Rothia aeria*	Shared	Shared	Periodontitis, dental infections	Rare cause of IE	(103) (104)
	*Rothia dentocariosa*	Shared	Shared	Periodontitis, caries	Rare cause of IE	(105) (106) (107)
	*Porphyromonas gingivalis*	Shared	Shared	Periodontitis, peri-implantitis	Correlated with cardiac disease	(108) (109) (110)
	*Escherichia coli*	Unique	Absent	Oral infection	Rare cause of IE	(111) (112)
	*Gemella sanguinis*	Absent	Absent	No known	Rare cause of IE	(113)
	*Candida spp.*	Shared	Shared	Caries, periodontitis, peri-implantitis	Rare cause of fungal IE, candidemia	(114) (115) (116) (117)

^a^ This table highlights the relationship between oral microorganisms implicated in IE, their presence in saliva and subgingival plaque samples, and their association with common oral pathologies, including dental caries, periodontitis, and peri-implantitis. “Shared” refers to microorganisms found in both saliva and subgingival plaque, while “Unique” indicates taxa exclusive to one sample type. These taxa have systemic implications, including contributions to infective endocarditis.

IE caused by *S. pneumonia,* which has ubiquitous pneumolysin genes with the *S. mitis* group, is rare and accounts for less than 3% of all cases, but can follow a severe clinical course (124). Risk factors for developing *S. pneumonia* IE include immunosuppression in the context of solid organ transplantation, malignancy or HIV infection, and chronic disorders, such as liver cirrhosis, COPD and diabetes mellitus (125). *S. pneumonia* releases pneumolysin and creates microlesions by forming large pores in cholesterol-containing membranes of eukaryotic cells (126). These microlesions can allow the formation of biofilms, which perpetuate further secretion of pneumolysins and deactivation of cardiac macrophages (125). It is currently unclear whether members of the *S. mitis* group possess this property, but this is likely based on multiple studies demonstrating that *S. mitis* species transfer genes in a similar manner to *S. pneumonia* (127, 128).

Periodontopathogens such as *Porphyromonas gingivalis* and *Fusobacterium nucleatum* have also been implicated in the development of IE (129, 130). These organisms can enter the bloodstream through bleeding gums and damaged periodontal tissues, potentially contributing to endocardial infections. *P. gingivalis*, in particular, has been strongly associated with cardiovascular diseases, including IE (131), due to its ability to evade the immune system and induce systemic inflammation. This pathogen is found in 85.75% of subgingival plaque samples from periodontal patients (132) and has even been isolated from cardiac valve specimens (133). However, other studies suggest that *P. gingivalis* may not directly influence the degeneration of aortic or mitral valves (131). Another bacterium, *Rothia dentocariosa*, commonly resides within the oral and respiratory tracts and is typically associated with dental caries or periodontal disease. Although *R. dentocariosa* is a rare cause of IE, it has been observed in patients with predisposing cardiac conditions and, in rare cases, in previously healthy individuals (134, 135).

### Oral microbiome interactions and its effect on periodontal and dental health in IE patients

The oral microbiome forms dental biofilms, which are a leading cause for the development of dental caries and periodontal diseases. Early phase colonisers, formed of predominantly *Streptococcus* species, can establish the right conditions for the population of disease-causing bacteria to grow at a later point (136). These later phase colonisers are usually anaerobic and, therefore, require early colonisers to extract oxygen from periodontal pockets to allow for their proliferation (137). The resulting biofilm is made up of proteins, polysaccharides and extracellular DNA, which promote interaction and population shifts between microbial species (138). Biofilm-associated microbes can survive in various stress conditions, being less susceptible to antibiotics and more likely to develop antibiotic resistance (139). Maintaining oral health based on regular oral hygiene measures, such as tooth brushing and flossing, helps to disturb these biofilms and reduce infections (140). However, if left undisturbed, the extracellular matrix characterising biofilms with multiple, surface-adherent microbial communities could favour the proliferation of more virulent species. This microbial shift could escalate into dysbiosis, a precursor of periodontal disease (141). Further research into the development of the biofilms initiated by *Streptococcal* species could have an impact on the antibiotic prophylaxis strategies in high-risk patients, which is increasingly important in the era of antimicrobial resistance.

Successful antibiotic prophylaxis is based on the principle that reducing bacteraemia during interventional procedures lowers the risk of IE (142). Early pre-clinical studies and observational data supported this strategy, leading to recommendations for the use of prophylactic antibiotics in high-risk cardiac patients undergoing bacteraemia-inducing procedures (143). However, the systematic use of prophylactic antibiotics has been contested due to the lack of randomised controlled trials (61). Previous meta-analyses demonstrated that antibiotic prophylaxis did not significantly reduce the risk of IE after dental procedures and that limiting its use to high-risk individuals did not increase the incidence of IE (144, 145). More recent evidence, however, indicates that antibiotic prophylaxis in high-risk patients undergoing invasive dental procedures, such as extractions and oral surgeries, significantly reduces the risk of IE (146–148). This conflicting evidence highlights the complex relationship between interventional procedures, bacteraemia, and IE, suggesting that successful prophylaxis depends on the clear identification of high-risk patients (149, 150).

Patients with a history of IE, prosthetic heart valves, and congenital heart disease have been defined as high-risk (according to (61)) and are recommended for antibiotic prophylaxis before invasive oral procedures (27). Some cardiac patients harbour significantly more oral Streptococci than non-cardiac patients (151), making them more susceptible to periodontal, peri-implant and other dental diseases, such as gingivitis and caries (152). Therefore, combining antibiotic prophylaxis with professional non-surgical periodontal therapy can reduce microbial load and inflammation, lowering the predisposition to IE. However, this approach must be balanced carefully, as antibiotics can disrupt the oral microbiota, leading to dysbiosis and promoting antibiotic resistance with long-term use (153). While short-term antibiotic use can eliminate pathogens, prolonged exposure may result in altered microbial diversity, negatively impacting both oral and systemic health (154). Furthermore, through resistome analyses, our group has identified a number of antibiotic resistance genes in saliva samples taken from IE patients (155).

Over the past years efforts have been made at Guy's and St. Thomas Trust (GSTT) to improve the understanding of the link between IE and oral health. A specific inpatient cardiac dental clinic has been set up to better inform patients of all ages who have, or are at significant risk of developing, IE. This has been coupled with an innovative oral health education programme for outpatients being seen in specialised valve outpatient clinics. It is possible that the physical and psychological burden of IE contributes to poor oral hygiene. Patients suffering from such chronic diseases may experience reduced motivation for self-care. Providing support through professional oral hygiene measures and behavioural interventions can improve outcomes. Our preliminary observations from these initiatives suggest that patient awareness as to the risks of IE with valvular heart disease is poor and that patients who present with IE tend to have suboptimal dental hygiene and higher rates of active periodontitis. Patients attending these initiatives are keen to improve oral hygiene after receiving professional oral hygiene instructions suggesting that the main barrier to improved oral health is a lack of awareness around the link to systemic disease. Observations such as these serve not only to reinforce the links between the oral microbiome and IE, but also to map out new collaborative pathways between cardiologists and dental teams with the aim of reducing the incidence and recurrence of IE in patients.

## Conclusion

The oral microbiome has been heavily implicated in the causation of IE. The current literature has identified key causative bacteria of IE, such as *S. aureus* and a series of *Streptococcus* species, as well as providing ideas for diagnostic improvements using metagenomic techniques. However, gaps still appear when looking at antibiotic strains and specific preventative steps that can be employed by high-risk individuals. Future studies should look to emulate past successes of metagenomic studies in order to identify biomarkers and microbial networks related to IE.
